# Open and closed distal anastomosis for acute type A aortic dissection repair: early and long-term outcomes from a contemporary series of 204 patients

**DOI:** 10.1186/1749-8090-10-S1-A314

**Published:** 2015-12-16

**Authors:** Pietro Giorgio Malvindi, Amit Modi, Szabolcs Miskolczi, Markku Kaarne, Theodore Velissaris, Clifford Barlow, Sunil K Ohri, Geoffrey Tsang, Steven Livesey

**Affiliations:** 1 Wessex Cardiothoracic Centre, University Hospital Southampton, Southampton, SO16 6YD, UK

## Background/Introduction

The current general consensus favours an open distal anastomosis for aortic dissection repair. A limited number of studies have compared the results between open and closed repair strategies.

## Aims/Objectives

We have reviewed our experience in the treatment of acute aortic dissection with open and closed distal anastomosis. We assessed the preoperative and intraoperative characteristics of the two cohorts of patients and analysed early and long-term survival, the neurologic outcomes and the evolution of the residual dissected aorta.

## Method

204 patients underwent repair of spontaneous acute type A aortic dissection between January 2000 and December 2013. Open and distal anastomosis strategies were equally used by all the first operators throughout the study period. Univariate comparisons of preoperative, operative and postoperative variables were performed between the two groups. Twenty-six variables were entered into a regression model to determine the impact on mortality and the occurrence of postoperative neurologic complications. The subgroup of patients with type 1 de Bakey and an intimal tear in the proximal aorta was studied with a similar analysis design. Mean FU was of 67 +/- 46 months. CT scan FU was available in 83 patients among survivors.

## Results

Patients in the open repair group were more likely to present and intimal tear located into the aortic arch and a dissection flap extending distally to the descending aorta. There were no differences in terms of mortality, morbidity and length of stay between the two groups of patients. Open repair with cerebral perfusion was associated with a lower rate of postoperative neurologic complications; DHCA alone was an independent risk factor for the occurrence of postoperative neurologic deficits. Patients who underwent an open distal anastomosis showed a significant higher rate of complete thrombosis of the false lumen (p = 0.036)

## Discussion/Conclusion

There is no difference in early and late survival between patients receiving an open distal repair and a closed anastomosis. The two group characterized different anatomical presentations of acute type A aortic dissection. The use of circulatory arrest with cerebral perfusion provided a reduced rate of postoperative neurologic deficits. The open repair was associated with a higher rate of complete false lumen thrombosis.

